# Molecular analysis of chondrocytes cultured in agarose in response to dynamic compression

**DOI:** 10.1186/1472-6750-8-71

**Published:** 2008-09-15

**Authors:** Carole Bougault, Anne Paumier, Elisabeth Aubert-Foucher, Frédéric Mallein-Gerin

**Affiliations:** 1 UMR 5086, CNRS, Université de Lyon, IFR 128, IBCP, Institut de Biologie et Chimie des Protéines, 7 passage du Vercors F-69367 Lyon FRANCE

## Abstract

**Background:**

Articular cartilage is exposed to high mechanical loads under normal physiological conditions and articular chondrocytes regulate the composition of cartilaginous matrix, in response to mechanical signals. However, the intracellular pathways involved in mechanotransduction are still being defined. Using the well-characterized chondrocyte/agarose model system and dynamic compression, we report protocols for preparing and characterizing constructs of murine chondrocytes and agarose, and analyzing the effect of compression on steady-state level of mRNA by RT-PCR, gene transcription by gene reporter assay, and phosphorylation state of signalling molecules by Western-blotting. The mouse model is of particular interest because of the availability of a large choice of bio-molecular tools suitable to study it, as well as genetically modified mice.

**Results:**

Chondrocytes cultured in agarose for one week were surrounded by a newly synthesized pericellular matrix, as revealed by immunohistochemistry prior to compression experiments. This observation indicates that this model system is suitable to study the role of matrix molecules and trans-membrane receptors in cellular responsiveness to mechanical stress. The chondrocyte/agarose constructs were then submitted to dynamic compression with FX-4000C™ Flexercell^® ^Compression Plus™ System (Flexcell). After clearing proteins off agarose, Western-blotting analysis showed transient activation of Mitogen-activated protein kinases (MAPK) in response to dynamic compression. After assessment by capillary electrophoresis of the quality of RNA extracted from agarose, steady-state levels of mRNA expression was measured by real time PCR. We observed an up-regulation of cFos and cJun mRNA levels as a response to compression, in accordance with the mechanosensitive character observed for these two genes in other studies using cartilage explants submitted to compression. To explore further the biological response of mouse chondrocytes to the dynamic compression at the transcriptional level, we also developed an approach for monitoring changes in gene transcription in agarose culture by using reporter promoter constructs. A decrease in promoter activity of the gene coding for type II procollagen, the most abundant protein in cartilage, was observed in response to dynamic loading.

**Conclusion:**

The protocols developed here offer the possibility to perform an integrated analysis of the molecular mechanisms of mechanotransduction in chondrocytes, at the gene and protein level.

## Background

Articular cartilage serves as a bearing surface for joints and is routinely exposed to mechanical loading. The mechanical properties of the tissue are due to its extracellular matrix particular composition. On the one hand, the load-bearing function is based on the high osmotic pressure created by the negatively charged glycosaminoglycans. These polyanions attract an excess of sodium ions into the tissue, resulting in an influx of water. On the other hand, the fibrillar collagen network, with type II collagen representing the major protein of cartilage, provides the tissue with its tensile strength. Thus, cartilage is a highly specialized tissue with relatively few cells, the chondrocytes, embedded in it. In this context, mechanical forces are thought to play an important role in regulating chondrocyte physiology. Several studies using cartilage explants or chondrocytes seeded in three-dimensional (3D) scaffolds have shown that mechanical compressive loading affects the chondrocyte metabolic activity [1-10]. However little is currently known regarding the biochemical pathways involved in mechanotransduction signalling in cartilage.

Numerous previous studies have examined the phenotypic responsiveness of chondrocytes to mechanical stress using the well-characterized experimental system consisting of isolated chondrocytes embedded within agarose hydrogels [11-15]. The ability of this 3D model system to maintain or re-establish the differentiation state of chondrocytes over extended culture periods has been extensively studied [16,17]. Also, of the 3D matrices, hydrogels may prove most suitable for analysis of mechanical response as they are homogeneous from the outset and fully surround cells embedded within. Additionally, the cell-agarose model could be used to examine the interactions of growth factors, such as Bone Morphogenetic Proteins, and mechanical stimuli on chondrogenic activity. Furthermore, it has been suggested that mechanical conditioning may be useful in cartilage-engineering context to stimulate in vitro biosynthesis by chondrocytes within 3D scaffolds prior to implantation. Considering that agarose or agarose/alginate hydrogels are clinically potential scaffolds for autologous chondrocyte implantation [18], the chondrocyte/agarose construct under compression is a model that could also provide insight into mechanotransduction within cartilage-engineered constructs.

Since the most recent studies indicate that the molecular mechanisms by which chondrocytes sense and respond to mechanical stimuli lead to changes at the levels of transcription, translation, post-translational modification, and matrix synthesis or degradation, there is a need to develop protocols to extract from agarose gels high quality RNA and proteins. We provide here general protocols for preparing and characterizing constructs of murine chondrocytes and agarose, and analyzing the influence of cell compression on the steady-state level of mRNA, gene transcription using reporter promoter construct, and signalling molecules phosphorylation state. The mouse model is of particular interest because of the availability of a large choice of bio-molecular tools suitable to study it, as well as genetically modified mice.

## Results and Discussion

### Preparation of chondrocyte/agarose constructs

**Figure 1:**
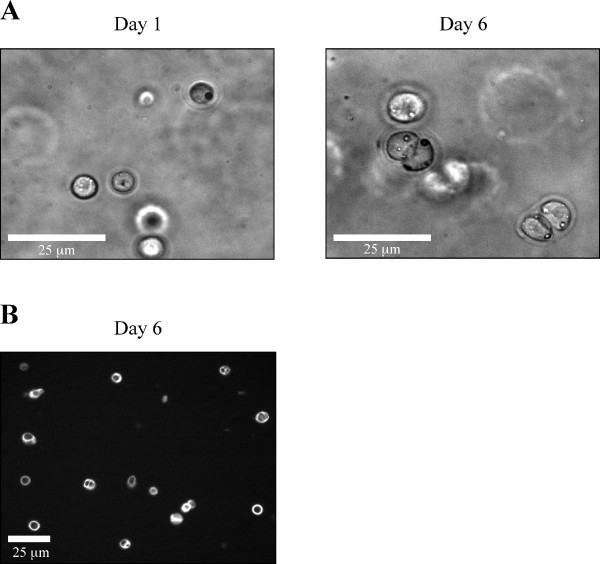
**Morphology and immunostaining of mouse chondrocytes embedded in agarose gel**. A) Chondrocytes cultured in 3D maintain their round morphology. Signs of cell division are observed. B) Pericellular staining with an anti-type II collagen antibody (image captured at the centre of the gel).

Embryonic mouse chondrocytes were isolated from the ventral parts of the rib cages of 17.5 dpc mice, as described [19,20]. Immediately after isolation, cells were embedded in agarose hydrogels with a cell density of 2 × 10^6 ^cells/mL of agarose gel. The final agarose concentration of chondrocyte/agarose constructs was 2%. After gelling, the cell/agarose constructs were punched into cylindrical gels to suit the 13 mm diameter foam ring of Biopress™ culture plates (Flexcell). Cells were maintained in culture for one week before compression. Since the presence of serum may hamper subsequent protein analysis, a progressive deprivation of serum was performed as detailed in Table 1. Besides, ascorbic acid was progressively added during this first week of culture to allow extracellular matrix deposition (Table 1). In these conditions, chondrocytes maintained their round morphology over one week as shown in Figure 1A. Signs of cell division were also observed, attesting the healthy behaviour of the cells in this culture system (Figure 1A). Moreover, pericellular matrix deposition was observed by immunostaining with an anti-type II collagen antibody after 6 days of culture (Figure 1B). Therefore, chondrocytes embedded in agarose hydrogel were surrounded by a newly synthesized extracellular matrix, prior to compression experiments. This observation indicates that this model system is suitable to study the role of matrix molecules and trans-membrane receptors in cellular responsiveness to mechanical stress. Regarding the potential involvement of cell deformation in mechanotransduction it should be added that elaboration of the pericellular matrix should be timely controlled and adapted to each cell type, agarose concentration and compression system that are used. For instance, previous studies have shown that elaboration of the pericellular matrix around bovine articular chondrocytes compressed within agarose gels protects the cells from morphological deformation [11,21].

**Table 1:** Timetable of progressive deprivation of serum over the 6-day culture period of chondrocyte/agarose constructs.

	**FBS**	**ITS**	**Ascorbic acid**	**Hepes buffer**
Day 0	10%	-	-	10 mM
Day 1	10%	-	-	10 mM
Day 2	5%	-	5 ng/mL	20 mM
Day 3	5%	1%	10 ng/mL	20 mM
Day 4	1%	1%	15 ng/mL	30 mM
Day 5	-	1%	20 ng/mL	30 mM
Day 6	-	1%	20 ng/mL	30 mM

Timetable of progressive substitution of ITS for serum in the culture medium of the chondrocyte/agarose constructs, over the 6-day culture period preceding compression. The concentrations of ascorbic acid and Hepes were progressively increased to allow recovery of the cells after their extraction.

### Choice of compression system

**Figure 2:**
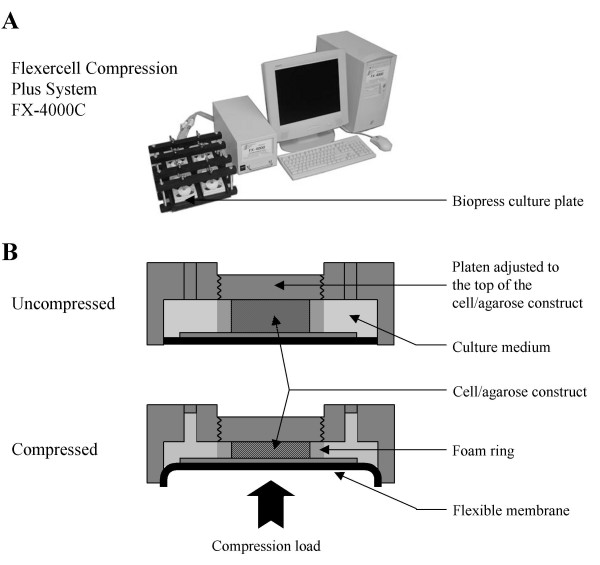
**Compression system**. A) FX-4000C™ Flexercell^® ^Compression Plus™ System (Flexcell International). A positive pressure compresses samples between a piston and stationary platen on the BioPress™ culture. B) Schematic diagram of the BioPress™ culture plate compression chamber in uncompressed or compressed position.

**Figure 3:**
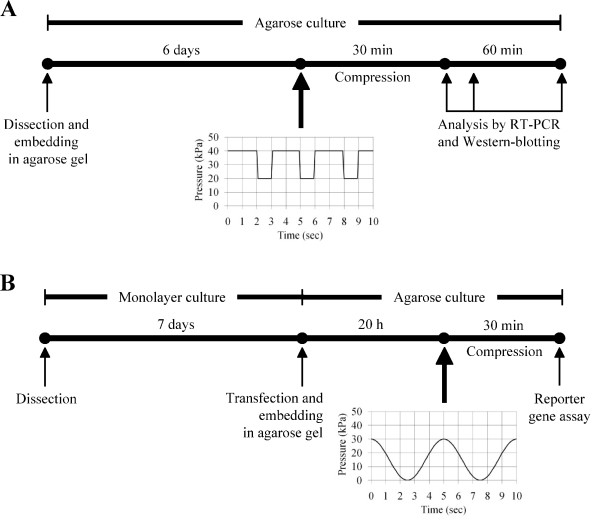
**General workflow of the analysis of the effect of compression**. A) Workflow scheme of the analysis of the effect of compression on gene expression and activation of signalling molecules in chondrocytes cultured in agarose. Immediately after their isolation from cartilage, chondrocytes are embedded in agarose gel. Cell/agarose constructs are cultured for 6 days to allow pericellular matrix deposition before being submitted to compression. At different time points after compression, total RNAs and proteins are isolated and analyzed by RT-PCR and Western blotting, respectively. B) Workflow scheme of the analysis of the effect of compression on gene promoter activity in chondrocytes cultured in agarose. After their isolation from cartilage, chondrocytes are first cultured in monolayer on plastic for one week, after which they are harvested and nucleofected with plasmids of interest. The transfected cells are embedded in agarose gels and the cell/agarose constructs are submitted to compression. After compression, the constructs are frozen in liquid nitrogen, lyophilized and lysed for reporter gene assay.

The mechanical stress that is applied on articular cartilage comprises a complex combination of strain, shear stress and compressive forces, the latter being more prevalent. Regarding investigation of chondrocyte mechanotransduction with the use of cell-scaffolds model systems, both static and dynamic compressions have been reported to influence chondrocyte differentiation or physiology. It should be kept in mind that the method of application, as well as the duration, frequency, and duty cycle of loading could modify biosynthetic response. In the method presented here, chondrocyte/agarose constructs were placed into individual wells of Biopress™ compression plates (Flexcell International) and we applied intermittent compression on these constructs by using the FX-4000C™ Flexercell^® ^Compression Plus™ System previously described [1] (Figure 2). The control samples were kept in unloaded conditions. Mechanical strain consisted of cyclical compression with pulses of 20 kPa (2 sec on, 1 sec off) superimposed on 20 kPa static offset pressure for 30 min (Figure 3A). This strain regimen was chosen here because it preserves agarose gel integrity needed to achieve correct transmission of the mechanical stress to the isolated cells embedded in it. Nevertheless, other types of compression devices can be used to study the effect of mechanical loading of chondrocytes embedded in agarose gels. With a more general view, the loading regimen should be adapted to agarose concentration.

### Protein extraction and analysis: Mechanical loading triggers MAPK activation

**Figure 4:**
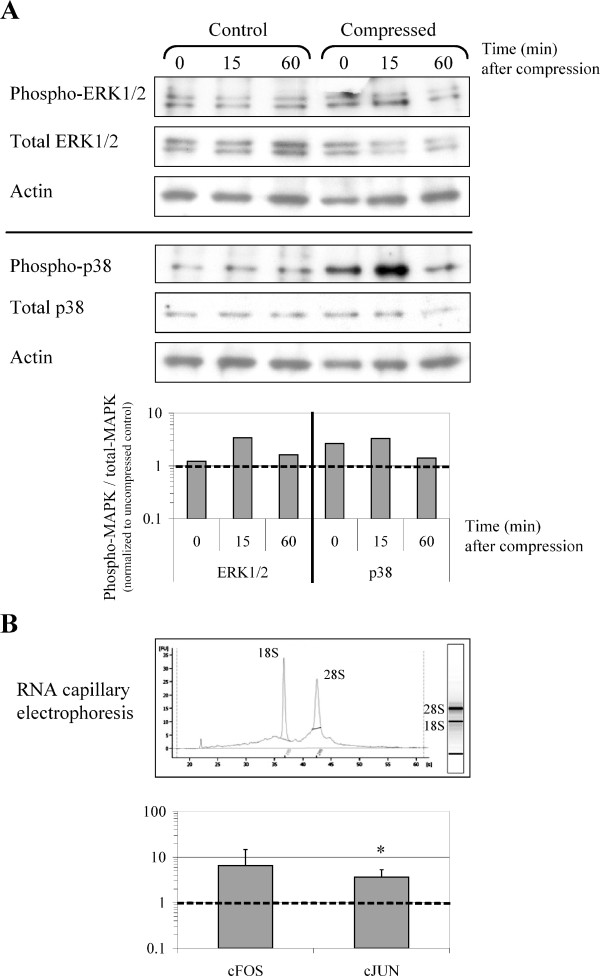
**Analysis of the molecular responses of chondrocytes cultured in agarose gels to dynamic compression**. Analysis of the responsiveness of chondrocytes in agarose gels immediately (0 min) or 15 min and 60 min after being subjected to dynamic loading at 0.33 Hz for 30 min. Control correspond to uncompressed cell/agarose constructs. A) Measurement of MAP kinases activation using Western-blotting analysis. A representative blot with quantification of phosphorylated ERK1/2 and p38 is shown. Mechanically-induced phosphorylation was normalized to uncompressed controls, as referred to the dashed line. Transient phosphorylation of ERK1/2 and p38 kinase is observed in response to dynamic compression. B) cFos and cJun mRNA levels are up-regulated in response to dynamic compression, as measured immediately after 30 min compression (time = 0 min after compression). Data are reported as means of three independent experiments with the standard deviations. The dashed line indicates the uncompressed control levels. * indicates significant difference between the compressed samples and the controls, p < 0.10. The electropherogram at the top shows the integrity of RNA used for RT-PCR analysis, as assessed by capillary electrophoresis with the Agilent 2100 Bioanalyzer.

At the different time points studied the chondrocyte/agarose constructs were frozen in liquid nitrogen and were then freeze-dried to concentrate proteins. Laemmli extraction buffer was added to freeze-dried gels and the samples were immediately boiled. To clear proteins off agarose, lysates were allowed to gel at room temperature, then transferred on paper filter to mini-spin columns and centrifuged. The exudates obtained were analyzed by Western blotting. By using such a protocol, we were able to detect transient activation of Mitogen-activated protein kinases (MAPK) in response to dynamic compression. For instance, transient phosphorylation of ERK1/2 and p38 were detected after dynamic loading (Figure 4A). This result is in accordance with previous studies using cartilage explants and showing that static or dynamic compression can induce the phosphorylation of ERK1/2 and p38 [22,23].

### RNA extraction and gene expression analysis: Mechanical loading stimulates cFos and cJun expression

Total RNAs from cell/agarose constructs were extracted using the RNeasy-mini kit (Qiagen). To facilitate agarose dissolution, QG buffer (Qiagen) was added to the classical RLT lysis buffer, and agarose was diluted in large volumes, as described in "Methods". According to the manufacturer's protocol, a DNase treatment was included to remove any contaminating genomic DNA. After assessment of the quality of RNA by capillary electrophoresis (Figure 4B), steady-state levels of mRNA expression was measured by real time PCR. We observed an up-regulation of cFos and cJun mRNA levels as an immediate response to compression (Figure 4B). Interestingly, it has already been shown that gene expression levels of cFos and cJun increase within 1 h of a variety of intact cartilage loading regimen [5,24,25]. It has also been shown that the binding of activating protein-1, the heterodimer of cFos and cJun, increases in response to dynamic compression of a tissue engineered construct [26]. Thus, the up-regulation of cFos and cJun mRNAs observed here in the chondrocyte/agarose constructs that have been submitted to compression is in agreement with the mechanosensitive character of these two genes.

### Analysis of human *COL2A1 *gene promoter activity in response to compression

To our knowledge, only two studies have attempted to monitor cartilaginous extracellular matrix gene transcription by chondrocytes in 3D culture in response to compression [3,27]. To explore further the biological response of embryonic mouse chondrocytes to the dynamic compression at the transcriptional level, we developed here an approach for monitoring changes in gene transcription in agarose culture by using reporter promoter constructs.

Specifically, we used chimeric plasmid "p3" (pGL2-0.387 kb) to monitor promoter activity of the human *COL2A1 *gene, which codes for type II procollagen [28]. "PGL2C" is a plasmid control carrying SV40 promoter instead of a fragment of *COL2A1 *promoter. These two plasmids are luciferase reporter vectors. We also used a plasmid encoding β-galactosidase under the control of Rous sarcoma virus (RSV) promoter to monitor transfection efficiency.

**Figure 5:**
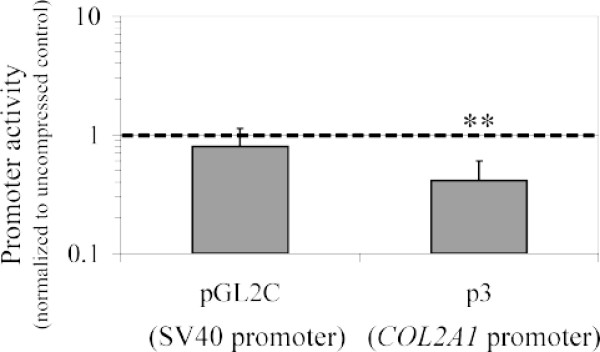
**Analysis of the human *COL2A1 *gene promoter activity in response to compression**. Analysis of the activity of the *COL2A1 *gene promoter in chondrocytes embedded in agarose gels immediately after the cell/agarose constructs were subjected to dynamic loading at 0.2 Hz for 30 min. A decrease in *COL2A1 *promoter activity in response to dynamic loading is observed in comparison with the control constructs that remained uncompressed. Data are reported as means of three independent experiments with the standard deviations. The dashed line indicates the uncompressed control levels. ** indicates significant difference between the compressed samples and the controls, p < 0.05.

After dissection, rib chondrocytes were first expanded on plastic during one week. This step of cell amplification was needed because nucleofection kills a high proportion of cells (see "Methods"), particularly freshly isolated chondrocytes. The cells were then trypsinized and nucleofected with the plasmids of interest. The transfected chondrocytes were embedded in agarose gels as described above and the cell/agarose constructs were placed in an incubator at 37°C in the presence of 5% CO_2 _overnight. In this case we did not allow the cells to build a pericellular matrix during a long pre-culture period in agarose since *COL2A1 *promoter activity is reduced after 24 h following transfection. The next day, the constructs were submitted to intermittent compression of 30 kPa using a sinusoid waveform at 0.2 Hz for 30 min. In the case presented here, the analysis of the activity of the *COL2A1 *gene promoter was performed immediately at the end of the compression regimen (workflow resumed in Figure 3B). A significant (p < 0.05) decrease in *COL2A1 *promoter activity in response to dynamic loading was observed in comparison with the control constructs that remained uncompressed (Figure 5). In the same vein, another study using bovine chondrocytes embedded in agarose gel has shown that dynamic loading decreases type II collagen promoter activity when measured 24 h after the end of the compression regimen [27].

## Conclusion

This study integrates protocols that allow analysis of the effects of compression on mouse chondrocytes embedded in agarose gels at the cellular, protein, and gene levels. Figure 3 summarizes the loading protocols we used for PCR, Western-blotting and gene promoter activity analyses but other types of loading regimen could be used for such analyses. The techniques presented here have been optimized so that the studies can be performed with chondrocytes isolated from a limited number of mouse embryos or new-borns and this point is important in regard of genetically modified mice. Besides, it is possible to apply these protocols to other types of chondrocytes, including human chondrocytes.

## Methods

### Preparation and Culture of chondrocyte/agarose constructs

Embryonic mouse chondrocytes were isolated from the ventral parts of the rib cages of 17.5 dpc mice. For each experiment, cells were pooled from all embryos from 2 or 3 OF-1 mouse litters. Mouse care and treatment were conducted in conformity with institutional guidelines in compliance with national and international laws and policies (Authorization n°69387416 given by the French Prefecture du Department du Rhone). Agarose hydrogels were prepared by mixing 2.5% low melting agarose (Seaplaque, Cambrex BioScience) with 5 × Dulbecco's modified Eagle's medium/Ham's F-12 (DMEM/F-12) containing 10% FBS, 50 mM Hepes and 500 U/mL Penicillin and 500 μg/mL Streptomycin (all products from Gibco). Cells were suspended in this agarose solution with a density of 2 × 10^6 ^cells/mL. 700 μL of the mixture was poured into wells of 24-well plates, and chondrocyte/agarose constructs were allowed to gel at room temperature. Constructs were then punched to obtain cylindrical gels (13 mm diameter and 3 mm height) that were placed into wells of Biopress™ compression plates (Flexcell international), within the 13 mm diameter foam ring (Figure 2). A volume of 4 mL DMEM/F-12 culture medium containing 10% FBS and 100 U/mL Penicillin and 100 μg/mL Streptomycin was added to each well. The cell/agarose constructs were maintained in culture for 6 days in 5% CO_2 _at 37°C. The culture medium was changed every day with a progressive deprivation of serum as detailed in Table 1.

### Compression

Samples were submitted to load with FX-4000C™ Flexercell^® ^Compression Plus™ System (Flexcell International). The strain regimen consisted either in (i) cyclical compression with pulses of 20 kPa at a frequency of 0.33 Hz (2 sec on, 1 sec off) superimposed on 20 kPa static offset pressure for 30 min, or in (ii) intermittent compression of 30 kPa using a sinusoid waveform at 0.2 Hz for 30 min, as indicated (Figure 3). As control, cell/agarose constructs were maintained under uncompressed conditions.

### Immunohistochemistry

The chondrocyte/agarose constructs were fixed in 4% formaldehyde for at least 24 h, after which they were embedded in Paraffin, sectioned at 7 μm, and then deparaffinised. Prior to antibody treatment, the sections were sequentially incubated with hyaluronidase (Sigma, type I-S, 800 U/mL) for 1 h at 37°C and 0.1% Triton X-100 for 20 min at room temperature. The sections were then incubated overnight with 2B1 anti-type II collagen primary mouse antibody (kind gift from Richard Mayne) [29] at 4°C. After washing in Phosphate Buffer Saline, the sections were incubated with Cy™2-conjugated AffiniPure donkey anti-mouse IgG (Jackson Immunoresearch) for 1 h at room temperature.

### Protein Extraction and Analysis

At the different time points studied the chondrocyte/agarose constructs were frozen in liquid nitrogen, freeze-dried and stored at -20°C until analysis. For protein extraction, 4 × Laemmli buffer was prepared (250 mM Tris-HCl, 20% glycerol, 10% SDS and bromophenol blue) and extemporaneously diluted to 1 × and supplemented with 3% 2-mercaptoethanol. A volume of 200 μL of this extraction buffer was added to each freeze-dried construct and the mixture was boiled immediately for 5 min. The lysates were allowed to gel at room temperature before being transferred on paper filter mini-spin columns (Pierce) and centrifuged at 12,000 g for 1 h at room temperature. The exudates were collected in mini-tubes and stored at -20°C until further analysis by Western blotting. The antibodies used in this study are described in Table 2.

**Table 2:** Antibodies used for Western blot analysis

**Target Protein**	**Antibodies**	**Source**
Actin	Actin (20–33) Antibody	Sigma #A-5060
p38 (total form)	p38 MAP kinase Antibody	Cell Signaling #9212
ERK1/2 (total form)	p44/42 MAP kinase Antibody	Cell Signaling #9102
p38 (phosphorylated form)	Phospho-p38 MAP kinase (Thr180/Tyr182) Antibody	Cell Signaling #9211
ERK1/2 (phosphorylated form)	Phospho-p44/42 MAP kinase (Thr202/Tyr204) Antibody	Cell Signaling #9101

### RNA Extraction

The protocol of RNA extraction was modified from the RNeasy-mini kit (Qiagen) procedure. Briefly, each chondrocyte/agarose construct was diced in a mixture of 1.5 mL QG buffer (Qiagen) and 2 mL RLT buffer (Qiagen kit) and the samples were kept at room temperature until complete dissolution, and then stored at -20°C. After defrosting, the samples were homogenized and supplemented with 2 mL 70% ethanol. The subsequent RNA purification steps were performed as described by the manufacturer. RNA quantity and quality were assessed by using a capillary electrophoresis system (RNA Nano kit and 2100 BioAnalyzer, Agilent).

### Reverse transcription and Real time PCR

Reverse transcription (RT) was performed as described [20]. For real time PCR amplification, a 20 μL reaction contained 1.3 μL of RT product, 10 μL SYBR green Supermix (Bio-Rad) in the presence of 300 nM of specific primers (see Table 3). Amplification was performed in an iCycler iQ (Bio-Rad), using the following conditions: an initial denaturation step of 2 min at 95°C, followed by 40 cycles of 30 sec at 95°C, 30 sec at 54°C, and an extension step of 3 min at 72°C. Levels of gene expression were determined by using the comparative *C*_t _method with *RPL13a *gene as endogenous control.

**Table 3:** Primers used for real time PCR analysis

**Genes**	**Primers**
RPL13a (reference gene)	Forward : atccctccaccctatgacaa
	Reverse : gccccaggtaagcaaactt
cFOS	Forward : gggacagcctttcctactacc
	Reverse : gatctgcgcaaaagtcctgt
cJUN	Forward : agggacccatggaagttttt
	Reverse : tttttctaggagttgtcagattcaaa

### Transfection

Prior to transfection, chondrocytes were amplified in monolayer culture for one week. After trypsinization, 3 × 10^6 ^cells were suspended in 100 μL of electroporation buffer and mixed with 4 μg plasmid of interest and 1 μg plasmid encoding β-galactosidase. The plasmids were transfected by using the Human Chondrocyte Nucleofector kit (Amaxa) according to the manufacturer's protocol (Program U-24). Although nucleofection leads to about 60% cell mortality, this method provides high transfection efficiency (around 80%) for the viable cells. This transfection efficiency was assessed by monitoring synthesis of green fluorescent protein in chondrocytes observed under a fluorescence microscope, after transfection of the corresponding expression vector (data not shown).

### Measure of *COL2A1 *promoter activity

Immediately after nucleofection, chondrocytes were embedded in agarose and the cell/agarose constructs were submitted to compression the following day. At the end of the compression regimen (20 h post-transfection), the constructs were frozen in liquid nitrogen and stored at -80°C. The frozen samples were then freeze-dried for 12 h before being grinded in TissueLyser (Qiagen) for 1 min at 20 Hz. The resulting powder was rehydrated in 200 μL of the kit lysis solution for 1 h at 4°C and reporter assays were performed using Dual-Light^® ^combined reporter gene assay system for detection of luciferase and β-galactosidase (Applied Biosystems), as described by the manufacturer, and MLX™ Microtiter^® ^plate luminometer (Dynex). *COL2A1 *promoter activity was obtained as a ratio of luciferase to β-galactosidase luminescence.

### Quantification and statistical analysis

For Western-blotting analysis, band intensity was quantified by densitometry using ImageQuant software (v5.2, Molecular Dynamics). The ratio of phospho-MAPK to total-MAPK band intensity was calculated and mechanically-induced phosphorylation was normalized to uncompressed controls. For the analysis of the compression effects on mRNA levels and promoter activity, data represent the mean and standard deviation values of 3 independent experiments. Statistical analysis was performed using a Student's paired, two-tailed *t*-test. Significance was reported at the 90% (*) or 95% (**) confidence level.

## Authors' contributions

CB and AP contributed to conception of the experiments and carried out most of them and CB helped to draft the manuscript. EAF participated in the design and analysis of protein extraction and carried out part of the protein assays. FMG conceived, coordinated the study and drafted the manuscript. All authors read and approved the final manuscript.
